# Alteration in Functional Magnetic Resonance Imaging Signal Complexity Across Multiple Time Scales in Patients With Migraine Without Aura

**DOI:** 10.3389/fnins.2022.825172

**Published:** 2022-03-07

**Authors:** Xiao Wang, Yutong Zhang, Wenchuan Qi, Tao Xu, Ziwen Wang, Huaqiang Liao, Yanan Wang, Jie Liu, Yang Yu, Zhenxi He, Shan Gao, Dehua Li, Guilin Zhang, Ling Zhao

**Affiliations:** ^1^College of Acupuncture, Moxibustion and Tuina, Chengdu University of Traditional Chinese Medicine, Chengdu, China; ^2^Hospital of Chengdu University of Traditional Chinese Medicine, Chengdu, China; ^3^Department of Neurology, Sichuan Provincial People’s Hospital, Chengdu, China

**Keywords:** brain activity dynamics, functional magnetic resonance imaging, brain complexity, migraine without aura, multiple time scales

## Abstract

**Background:**

Migraine is a primary neurological disorder associated with complex brain activity. Recently, mounting evidence has suggested that migraine is underpinned by aberrant dynamic brain activity characterized by linear and non-linear changes across a variety of time scales. However, the abnormal dynamic brain activity at different time scales is still unknown in patients with migraine without aura (MWoA). This study aimed to assess the altered patterns of brain activity dynamics over different time scales and the potential pathophysiological mechanisms of alterations in patients with MWoA.

**Methods:**

Multiscale entropy in 50 patients and 20 healthy controls (HCs) was calculated to investigate the patterns and altered brain complexity (BC) across five different time scales. Spearman rank correlation analysis between BC in regions showing significant intergroup differences and clinical scores (i.e., frequency of migraine attacks, duration, headache impact test) was conducted in patients with MWoA.

**Results:**

The spatial distribution of BC varied across different time scales. At time scale1, BC was higher in the posterior default mode network (DMN) across participants. Compared with HCs, patients with MWoA had higher BC in the DMN and sensorimotor network. At time scale2, BC was mainly higher in the anterior DMN across participants. Patients with MWoA had higher BC in the sensorimotor network. At time scale3, BC was mainly higher in the frontoparietal network across participants. Patients with MWoA had increased BC in the parietal gyrus. At time scale4, BC is mainly higher in the sensorimotor network. Patients with MWoA had higher BC in the postcentral gyrus. At time scale5, BC was mainly higher in the DMN. Patients with MWoA had lower BC in the posterior DMN. In particular, BC values in the precuneus and paracentral lobule significantly correlated with clinical symptoms.

**Conclusion:**

Migraine is associated with alterations in dynamic brain activity in the sensorimotor network and DMN over multiple time scales. Time-varying BC within these regions could be linked to instability in pain transmission and modulation. Our findings provide new evidence for the hypothesis of abnormal dynamic brain activity in migraine.

## Introduction

Migraine is a debilitating neurological disorder associated with brain excitability dysfunction characterized by attacks of moderate or severe unilateral throbbing and pulsating headache (Ashina, 2020). The suggested mechanism of migraine is the dysfunction of the brain in regulating external stimuli and pain (Kuner and Kuner, 2021). Accumulating evidence shows that normal brain function is based on time-varying linear and non-linear information exchanges (Breakspear, 2017; Lim et al., 2021). Thus, it is essential to clarify functional brain abnormalities present over different time scales in migraine and how they are associated with migraine attack severity. Existing studies have focused on exploring the brain mechanisms of migraine using functional magnetic resonance imaging (fMRI).

Some fMRI studies have captured the differences between migraine and healthy controls (HC) across different spatial scales. For example, Ning et al. (2017) used the amplitude of low-frequency fluctuation (ALFF) to analyze fMRI data and found decreased ALFF values in the left calcarine, cuneus, and parietal gyrus, and increased ALFF values in the right hippocampus, parahippocampal gyrus, insula, middle temporal gyrus, and superior temporal gyrus. Network-based analyses suggest that abnormalities of the brain in migraine mainly affect the default mode network (DMN), sensorimotor network, frontal-parietal network, and limbic system (Kathleen et al., 2010; Yang M. X. et al., 2014; Zou et al., 2019). Accumulating evidence indicates that the pathophysiology of migraine is based on abnormal brain dynamics (Liu et al., 2017; Tu et al., 2019, 2020; Yiheng et al., 2021). For example, by using independent component-based network analyses, Tu et al. (2019) revealed abnormal thalamocortical network dynamics in migraine. Lim et al. (2021) revealed changes in fMRI signal variability in migraines. However, the time-varying characteristics of abnormal brain dynamic activity in migraineurs have not been well studied.

Traditionally, brain dynamic activity has been mainly explored based on measuring the mean or variance of the sequence (Chen et al., 2021; Lim et al., 2021) in a single time scale. These methods often overlooked the importance of the interaction among numerous neuronal circuits over a wide range of temporal and spatial scales, which is the basis of the brain to adapt to the ever-changing environment and to perform various mental functions (Yang et al., 2015; Wang et al., 2019). To broaden the spatio-temporal understanding of the brain, the brain complexity (BC) method was proposed (Costa et al., 2002, 2005). Based on the fusion of the multi-scale sampling method and sample entropy, the multi-scale sample entropy (MSE) method provides a profile of entropy over multiple time scales to explore the non-linear dynamic characteristics of brain activity. Further, multiple time scales BC has been found altered in many brain disorders, including attention deficit hyperactivity disorder (Sokunbi et al., 2013), schizophrenia (Yang et al., 2015; Wang et al., 2017a), and Alzheimer’s disease (Wang B. et al., 2017). Recently, several studies conducted with neuroimaging and EEG techniques have shown that migraine is accompanied by changes at multiple time levels in the brain (de Tommaso, 2019; Lim et al., 2021). Altogether, these studies demonstrated the potential of BC as a biomarker for various brain disorders, including migraine.

Based on previous evidence, we hypothesized that patients with migraine may show different patterns of abnormal brain activity dynamics over different time scales. To test our hypothesis, we first identified time scale-specific spatial distributions of BC using a one-sample *t*-test within different groups. We then utilized two sample *t*-tests between the migraine without aura (MWoA) and healthy control (HC) groups over different time scales to explore the altered brain activity dynamics. Finally, we performed a correlation analysis to explore the relationship between aberrant and clinical symptoms to test the contributions of BC alterations to the clinicopathology of migraine.

## Materials and Methods

### Participants

Fifty patients with MWoA were enrolled from the outpatient clinic of the Departments of Neurology in two clinical centers: (1) the Hospital of Chengdu University of Traditional Chinese Medicine; and (2) Sichuan Provincial People’s Hospital. Patients were enrolled in the study from August 2018 to April 2020. Twenty healthy controls were recruited from Chengdu University of Traditional Chinese Medicine and communities in Chengdu. All participants were scanned in the Huaxi MR Research Center, Sichuan University, China.

The diagnosis of MWoA was established according to the ICHD III-beta criteria (Headache Classification Committee of the International Head, 2018). Participants who met all the following inclusion criteria were included in the study: (1) female, 18–50 years old, right-handed; (2) fulfilling criteria for migraine without aura; (3) history of migraine without aura for 12 months or more; (4) did not receive acupuncture treatment or other preventive treatment within the last 3 months; and (5) no long-term use of analgesics. Patients and healthy controls with any of the following conditions were excluded: (1) macroscopic T2-visible brain lesions on magnetic resonance imaging (MRI) scans; (2) existence of additional psychiatric or neurological disorders; (3) taking any drugs affecting the central nervous system; (4) no current or previous antipsychotic medication, immunodeficiency, bleeding disorders, or allergies; (5) MRI contraindications or claustrophobia; and (6) alcohol or drug abuse. For the HC, they should either have no personal or family history of migraine or other headaches. This study was approved by the Ethics Committee of the Hospital of Chengdu University of Traditional Chinese Medicine and was conducted following the Declaration of Helsinki (World Medical Association, 2013). Voluntary written informed consent was obtained from each subject after verbal and written explanation of the study.

### Outcomes Measures

The clinical outcome measures were the change in the frequency of migraine attacks, duration, and headache impact test (HIT-6). In addition, migraine-specific quality-of-life questionnaires (MSQ) were assessed.

### Functional Magnetic Resonance Imaging Acquisitions

All subjects were instructed to rest with their eyes closed, not to think of anything in particular, and not to fall asleep during the scan. fMRI data were acquired with a Siemens Trio 3.0 Tesla MRI system equipped with a high-speed, eight-channel, phased-array head coil. The functional images were collected transversely using an echo-planar imaging (EPI) sequence with the following settings: TR/TE = 2,000 ms/30 ms, flip angle = 90°, 30 slices, 64 × 64 matrix, field of view = 240 × 240 mm^2^, interslice gap = 0 mm, and voxel size = 3.75 × 3.75 × 5 mm^3^. For each subject, 180 functional volumes were obtained.

All patients had been free from a typical migraine attack for at least 1 week before the MRI scan. The scan would be postponed to ensure that the migraine patient is in the interval of migraine attack at the time of the scan. Further, after scanning, all participants reported that they did not experience any headaches or migraines and remained awake during the measurement.

### Functional Magnetic Resonance Imaging Data Preprocessing

Resting-state fMRI images were preprocessed using the toolbox for Data Processing and Analysis of Brain Imaging (DPABI^1^) (Chao-Gan and Yu-Feng, 2010). The first 5 volumes were discarded to avoid the non-equilibrium effects of magnetization, and slice timing and realignment correction were performed for the remaining images. Any participant with maximum head movement greater than 2.0 mm translation or more than 2.0° rotation was not included. Data were further normalized to the EPI template (resampled voxel size of 3 × 3 × 3 mm). Then, several covariates including Fristion 24 motion parameters, the cerebrospinal fluid, and white matter signals were regressed as nuisance variables to reduce spurious variance. No global signal regression was performed to avoid introducing distortion into the time series data (Yang G. J. et al., 2014). Afterward, detrending and band-pass filtering (0.01–0.08 Hz) were conducted. Finally, given that resting-state activity is sensitive to minor head movement, we calculated the mean frame-wise displacement (FD) to further determine the comparability of head movement across groups (HC: 0.13 ± 0.04; MWoA: 0.14 ± 0.06; mean ± SD, *p* = 0.43). “Bad” time points (FD > 0.5 mm), as well as their one-back and two-forward time points, were then scrubbed and interpolated by spline interpolation (Power et al., 2012).

### Analysis of Brain Complexity

**FIGURE 1:**
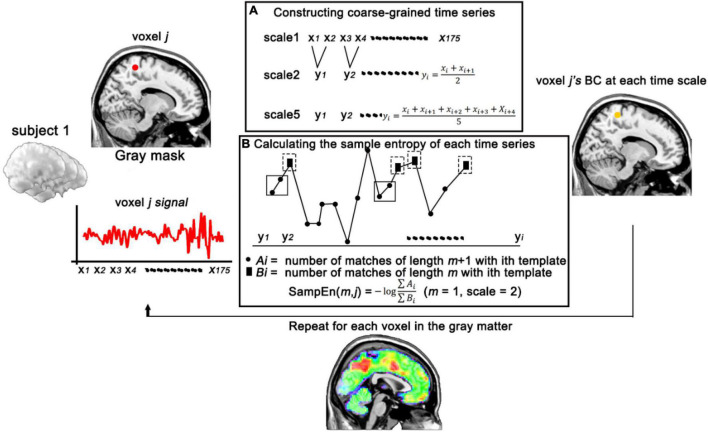
Schematic illustration of the calculation of BC. **(A)** Constructing coarse-grained time series of voxel *j* in different time scales. **(B)** Calculating the sample entropy of voxel *j* (with *m* = 1, scale = 2) at each time series. BC, brain complexity.

**FIGURE 2:**
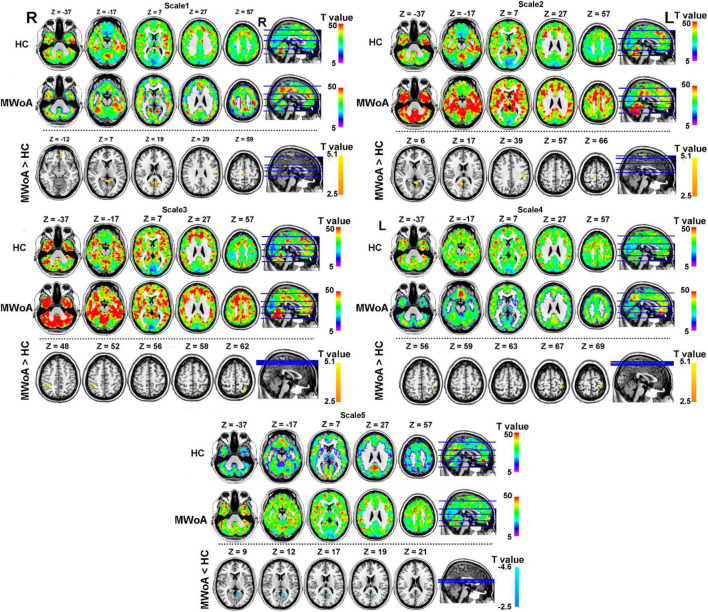
Spatial distribution of BC at each group and altered of BC in MWoA group at each time scale compared with HC group (GRF correction at voxel significant *p* < 0.005, cluster significant *p* < 0.05). The part above the dotted line describes the BC distribution for each group at different time scales. The warm color represents higher BC and the cool color represents lower BC in the brain. The part under the dotted line describes the abnormal BC in the MWoA group, compared with HC. The warm color represents the higher BC in the MWoA group and the cool color represents the lower BC in the MWoA group, compared with the HC group. MWoA, patients with migraine without aura; HC, healthy controls; L, left; R, right.

BC is calculated from the MSE method. MSE analyses were developed as a biologically meaningful measure of complexity (Costa et al., 2005). Sample entropy is used in MSE analysis because it provides greater consistency and is less dependent on a given signal length compared with other entropy methods (Richman and Moorman, 2000). MSE calculation can be briefly summarized in three steps: (1) multiscale sampling and constructing coarse-grained time series according to different scales (Figure 1); (2) calculating the BC, the sample entropy of each time series (Figure 1); the BC was obtained by Eq. (1):


(1) $$B\,C\,\left(m,r,N\right)=-l\,o\,g\,\frac{C^{m+1}\,\left(r\right)}{C^{m}\,\left(r\right)}$$

where *m* is the pattern length, *r* (similarity factor) represents a proportion of the standard deviation (SD) of the signal series is a distance threshold, and *N* is the length of the signal sequence. *C*^*m*^(*r*) (Figure 1, ∑ *B*_*i*_) is the sum which measures the average likelihood of *m*-length patterns in a signal series; *C*^*m* + 1^(*r*) (Figure 1, ∑ *A*_*i*_) is the sum which measures the average likelihood of *m+1*-length patterns in a signal series. Two patterns match if the distance is less than the tolerance of *r*. (3) comparing the sample entropy over a range of scales. Here, we used the previous parameters for MSE calculation that *m* = 1 and *r* = 0.35 and scale factors up to 5 (Yang et al., 2015; Wang et al., 2017b). To further confirm the effects of sequence length on sample entropy, 1,000 random splices were performed on the mean sequences of all participants (with 50–500 time points), and then 2-scale sample entropy was calculated (Supplementary Figure 1). Results revealed that the influence of sequence length on sample entropy does exist and gradually weakens with the increase of sequence length.

### Statistical Analysis

Statistical analysis of the BC was conducted using MATLAB. One-sample *t*-tests were utilized to assess the time-specific abnormal spatial distribution of BC in each group. Regional differences between migraines and HCs were examined using two-sample *t*-test at each scale. Gaussian random field (GRF) corrections (with voxel *p* < 0.005, cluster *p* < 0.05) were conducted for the comparisons of five scales (Han et al., 2021). GRF correction was conducted using the GRF program in DPABI software (see text footnote 1). Brain regions showing significant differences based on the results of two-sample *t*-test during the above analysis were defined as regions of interests (ROIs) for the following analysis. ROIs were defined as 4-mm spheres with a center at the peak position of statistical difference. Correlation analysis was then performed between the mean BC in the ROIs and the clinical symptoms of the MWoA patients.

## Results

### Demographics and Clinical Symptoms

Fifty patients were well matched with twenty HCs. The demographic characteristics of the participants are given in Table 1.

**TABLE 1:** Demographic and clinical characteristics.

Demographics Mean (*SD*)	HC *N* = 20	MWoA *N* = 50	*p*-value
Age (Year)	36.3 (6.2)	36.5 (9.76)	0.93^a^
Gender (Male/Female)	10/10	18/32	0.28^b^
Handedness (Right/Left)	20/0	50/0	—
Duration (Years)	—	10.3 (7.5)	—
Frequency of attack (d/m)	—	6.6 (4.4)	—
Average duration of a migraine attack (h)	—	12.6 (13.7)	—
Headache days	—	10.1 (8.8)	—
VAS score	—	5.8 (1.9)	—
HIT6 score	—	61.9 (6.9)	—
MSQ-function limitation	—	57.8 (18.3)	—
MSQ-function disorder	—	71.9 (19.8)	—
MSQ-emotion	—	73.5 (21.8)	—

*^a^p-value was obtained by two-sample t-test between HC and MWoA. ^b^p-Value was obtained by χ^2^ two-tailed test among three groups. HC, healthy controls; MWoA, patients with migraine without aura; SD standard deviation; VAS, visual analog scale; HIT-6, headache impact test-6; MSQ, migraine-specific questionnaire.*

### Spatial Distribution of Brain Complexity at Different Time Scales Within Each Group

The spatial distribution of BC at each group is shown in Figure 2 (above the dotted line). The topography of BC in the MWoA and HC groups showed a non-uniform distribution. In time scale1, BC was mainly higher in the precuneus, superior frontal gyrus, and parietal gyrus across subjects. In time scale2, BC was mainly higher in the medial orbitofrontal, temporal gyrus, and parietal gyrus across subjects. In time scale3, BC was mainly higher in the frontal-parietal regions across subjects. In time scale4, BC was mainly higher in the parietal gyrus. In time scale5, BC was mainly higher in the medial frontal gyrus and precuneus (GRF correction: voxel significant *p* < 0.005, cluster significant *p* < 0.05).

### Alteration of Brain Complexity in Migraine Without Aura at Different Time Scales

To test our hypotheses, we computed T-maps comparing the MWoA group with the HC group at different time scales (Table 2). The results revealed that in the time scale1, patients with MWoA showed increased BC in the left precuneus, left medial orbitofrontal, postcentral gyrus, and paracentral lobule (GRF: voxel significant *p* < 0.005, cluster significant *p* < 0.05). In time scale2, patients with MWoA showed increased BC in the right precuneus, left postcentral gyrus, and left paracentral lobule (GRF correction: voxel significant *p* < 0.005, cluster significant *p* < 0.05). In time scale2, patients with MWoA showed increased left superior parietal gyrus and right parietal gyrus (GRF correction: voxel significant *p* < 0.005, cluster significant *p* < 0.05). In time scale4, patients with EOS showed increased BC in the left postcentral gyrus (GRF correction: voxel significant *p* < 0.005, cluster significant *p* < 0.05). Finally, In time scale5, patients with MWoA showed reduced BC in the left precuneus (GRF correction: voxel significant *p* < 0.005, cluster significant *p* < 0.05) (see Figure 2 below the dotted line).

**TABLE 2:** Alteration of BC in MWoA at different time scales compared with the HC group.

Scale/group MWoA vs. HC	Brain areas	L/R	Cluster size voxels	*T-*value	Peak coordinate (MNI)		
					X	Y	Z
Scale1 MWoA > HC	Precuneus	L	205	5.16	−6	−45	6
	Medial orbitofrontal	L	36	4.18	−6	51	−12
	Postcentral gyrus	L	50	4.13	−39	−24	39
	Paracentral lobule	L	54	3.90	0	−24	63
Scale2 MWoA > HC	Precuneus	R	134	5.23	−6	−47	9
	Paracentral lobule	L	57	4.06	−3	−24	66
	Postcentral gyrus	L	51	3.87	−36	−23	36
Scale3 MWoA > HC	Superior parietal gyrus	L	35	3.99	−36	−54	60
	Inferior parietal gyrus	R	40	4.63	36	−42	48
Scale4 MWoA > HC	Postcentral gyrus	L	51	3.69	−39	−39	57
Scale5 MWoA < HC	Precuneus	L	62	−4.71	−12	−57	12

*BC, brain complexity; HC, healthy controls; MWoA, patients with migraine without aura; L, left; R, right.*

### Relationships Between Brain Complexity and Clinical Symptoms

**FIGURE 3:**
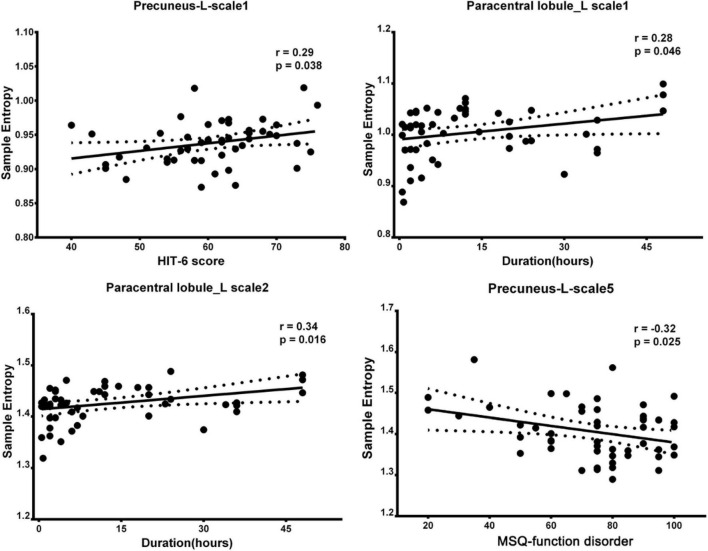
Correlation between regions showed altered BC and clinical scale of symptoms in patients with MWoA (*p* < 0.05, uncorrected). BC, brain complexity; MWoA, patients with MWoA; L, left; R, right; HIT-6, headache impact test; MSQ, migraine-specific questionnaire.

Spearman rank correlation was calculated between the BC in the ROIs at different time scales and clinical symptoms scores. In Figure 3, the left precuneus with increased BC is significantly positively correlated with the HIT-6 scores, and the left paracentral lobule with increased BC is significantly positively correlated with the duration in time scale1. In time scale2, the left paracentral lobule with increased BC is significantly positively correlated with the duration. In time scale5, the left precuneus with reduced BC is significantly negatively correlated with the MSQ-function disorder scores.

## Discussion

To the best of our knowledge, this is the first study to investigate the non-linear properties of brain dynamics over different scales and their alterations in patients with MWoA. We identified the topography patterns of BC over five different time scales. Patients with MWoA showed specific differences from the HC group at each time point. Compared with the HC group, patients showed increased BC (increased sensitivity) in the precuneus, frontal cortex, and sensorimotor cortex over time scales 1–4 and decreased BC in the precuneus over time scale5. Differences in the paracentral lobule and precuneus were significantly correlated with clinical symptoms. Our findings shed light on multiscale abnormalities in brain activity dynamics in migraine and its relevance to clinical symptoms.

### Spatial Topography of Brain Complexity in Migraine Without Aura Group Over Different Time Scales

Different and non-uniform distributions of BC topography were found in the HC and MWoA groups. Across all time scales, the precuneus and frontal gyrus had significantly higher BC than the HC group. These brain regions are consistent with those found by linear and single-time scale methods (de Tommaso, 2019; Lim et al., 2021). Higher variability in these regions suggests the abnormal coding of headaches in migraineurs (Bosma et al., 2018; Rogachov et al., 2018). Moreover, these findings implied that the precuneus and frontal gyrus are involved in the regulation of brain activity over multiple time scales, which are in line with the previous studies (Wang et al., 2017b; Lim et al., 2021). Notably, the BC in patients with migraine over time scale5 is different from that over the other time scales. The likely reason is that longer time scales capture lower frequencies of brain activity (Gohel and Biswal, 2015; Jiang et al., 2015), which suggests remote dysregulation of information in the brain of patients with migraine. Our findings suggest the abnormalities of the brain across multiple temporal and spatial scales in migraine.

### Altered Brain Complexity Pattern in Patients With Migraine Without Aura at Different Time Scales

Time-scale specific differences between the MWoA and HC groups were detected. Over time scale1, patients with MWoA showed increased BC in the left precuneus, left medial frontal orbital, left postcentral gyrus, and left paracentral lobule. Over time scale2 and time scale4, the abnormalities in BC were mainly found in the sensorimotor network. Over time scale3, the abnormalities in BC were mainly found in the frontoparietal joint area. Over time scale5, the abnormalities in BC were mainly found in the DMN. The precuneus is considered the center of a wide spectrum of highly integrated tasks (Cavanna and Trimble, 2006), such as the integration of auditory, somatosensory, and visual information, and the perception and transmission of pain (Zhe et al., 2021). Its dysfunction has been widely found to be associated with clinical manifestations of migraine (Tu et al., 2019). The medial orbitofrontal, as the key node in the DMN, is involved in the cognitive aspects of pain processing and top-down modulation of pain (Schwedt et al., 2015). Our results provide more detail on the DMN anomaly hypothesis of migraines (Tessitore et al., 2013; Zou et al., 2019; Chen et al., 2021) and that these abnormalities vary over time in migraineurs. The postcentral gyrus, paracentral lobule, and parietal gyrus are important parts of the sensorimotor network (Quairiaux et al., 2011), which is closely related to pain and is the main pain-regulating central system (Bornhovd et al., 2002; Liu et al., 2021). The current findings are in line with those of previous studies that found that migraine is associated with abnormalities in the pain perception (Russo et al., 2018) and processing system (Coppola et al., 2020). Interestingly, the current study showed that BC in the patient’s precuneus was elevated over time scale1 but reduced over time scale5. This is a new finding compared with the previously reported dynamic analyses in patients with migraine, which suggests that the precuneus abnormality spans multiple time scales (Gohel and Biswal, 2015; Wang et al., 2017a). Our findings provide new evidence of brain dynamics for the large-scale network anomaly hypothesis (Cai et al., 2018) in migraine.

### Correlation With Clinical Symptoms

To better explore the possible physiological mechanisms underlying altered BC in migraine, a correlation analysis was also performed. We noted that clinical symptoms were associated with BC values in the paracentral lobule and precuneus. The paracentral lobule is an important part of the pain matrix and directly accepts pain signals (May, 2006). Abnormalities in the precuneus are commonly reported to correlate with migraine frequency (Zhao et al., 2017; Tu et al., 2019). The current findings suggest that the BC in these regions is progressively associated with the severity of the clinical symptoms of migraine. Our findings suggest that the DMN and sensorimotor network showed altered excitability in the form of complexity during the resting state, which may contribute to the severity of headaches.

### Limitation

Some limitations should be considered in the present study. Firstly, the data length used in this study is only 175 time points. Although BC is considered independent of data length (Shi et al., 2020), the effect of sequence length still needs to be noted over short time series. Secondly, the proportion of men and women in this study is not significant between groups, but there is an imbalance. The impact of gender should be considered in future studies. Thirdly, the sample size used in this study is relatively small, which limits the statistical power of our results. The small sample size weakens the correlation in this paper, which also limits the reliability of research results. Larger sample size is necessary to confirm the results of the current study.

## Conclusion

Our findings reveal altered brain activity dynamics in patients with migraine across multiple time scales measured using a novel method. These findings support the hypothesis of abnormal brain dynamics and provide details of non-linear anomalies of brain activity dynamics in patients with migraine. Moreover, the altered BC in the precuneus and paracentral lobule was associated with clinical symptoms, suggesting that the symptoms were related to abnormal brain dynamics. Our observations may provide novel insights into the pathophysiological mechanisms underlying migraine.

## Data Availability Statement

The raw data supporting the conclusions of this article will be made available by the authors, without undue reservation.

## Ethics Statement

The studies involving human participants were reviewed and approved by the Ethics Committee of the Hospital of Chengdu University of Traditional Chinese Medicine. The patients/participants provided their written informed consent to participate in this study.

## Author Contributions

LZ, DL, and YW designed the study and conceptualized the protocol for healthy subjects. YZ, TX, and ZW adapted this protocol for patients with migraines without aura and evaluated them. WQ, HL, JL, and YY managed the literature searches and analyses. ZH, SG, and GZ undertook the statistical analyses. XW wrote the first draft of the manuscript. All authors contributed to and have approved the final manuscript.

## Conflict of Interest

The authors declare that the research was conducted in the absence of any commercial or financial relationships that could be construed as a potential conflict of interest.

## Publisher’s Note

All claims expressed in this article are solely those of the authors and do not necessarily represent those of their affiliated organizations, or those of the publisher, the editors and the reviewers. Any product that may be evaluated in this article, or claim that may be made by its manufacturer, is not guaranteed or endorsed by the publisher.

## Footnotes

1
http://rfmri.org/DPABI
