# Supplementary material for: Alteration in Functional Magnetic Resonance Imaging Signal Complexity Across Multiple Time Scales in Patients With Migraine Without Aura
Source: Front Neurosci. 2022 Mar 7;16:825172. doi: 10.3389/fnins.2022.825172 (PMC8957082; doi:10.3389/fnins.2022.825172)
Supplement: Supplementary file 1 [file Data_Sheet_1.docx]

**Alteration infunctional magnetic resonance imaging signal complexity across multiple time scales in patients with migraine without aura**

**Xiao Wang^1^, Yutong Zhang^1^, Wenchuan Qi^1^, Tao Xu^1^, Ziwen Wang^1^, Huaqiang Liao^2^, Yanan Wang^1^, Jie Liu^3^, YangYu^1^, Zhenxi He^1^, Shan Gao^1^, Dehua Li^2^, Guilin Zhang^1^, Ling Zhao^1,*^**

^1^College of Acupuncture, Moxibustion and Tuina, Chengdu University of Traditional Chinese Medicine, 37 Shi’erQiao Rd, Chengdu 610075, Sichuan, China

^2^Affiliated Hospital of Chengdu University of Traditional Chinese Medicine, 39 Shi’erQiao Rd, Chengdu 610072, Sichuan, China

^3^Department of Neurology, Sichuan Provincial People's Hospital.

*Corresponding author: Ling Zhao, Chengdu University of Traditional Chinese Medicine, No. 37 Shi’erQiao Rd, Chengdu, Sichuan 610075, China. Email: [zhaoling@cdutcm.edu.cn](mailto:zhaoling@cdutcm.edu.cn)

**S1 Effects of sequence length on sample entropy**

BC is a data-driven voxel-based approach, which is largely independent of data length and displays relative consistency over a broader range of possible parameters(Shi et al., 2020).To further confirm the effects of sequence length on our results, 1000 random splices were performed on the mean sequences of all participants (with 50-500 time points), and then 2-scale sample entropy was calculated(supplements sFigure1).The influence of sequence length on sample entropy does exist and gradually weakens with the increase of sequence length.

**
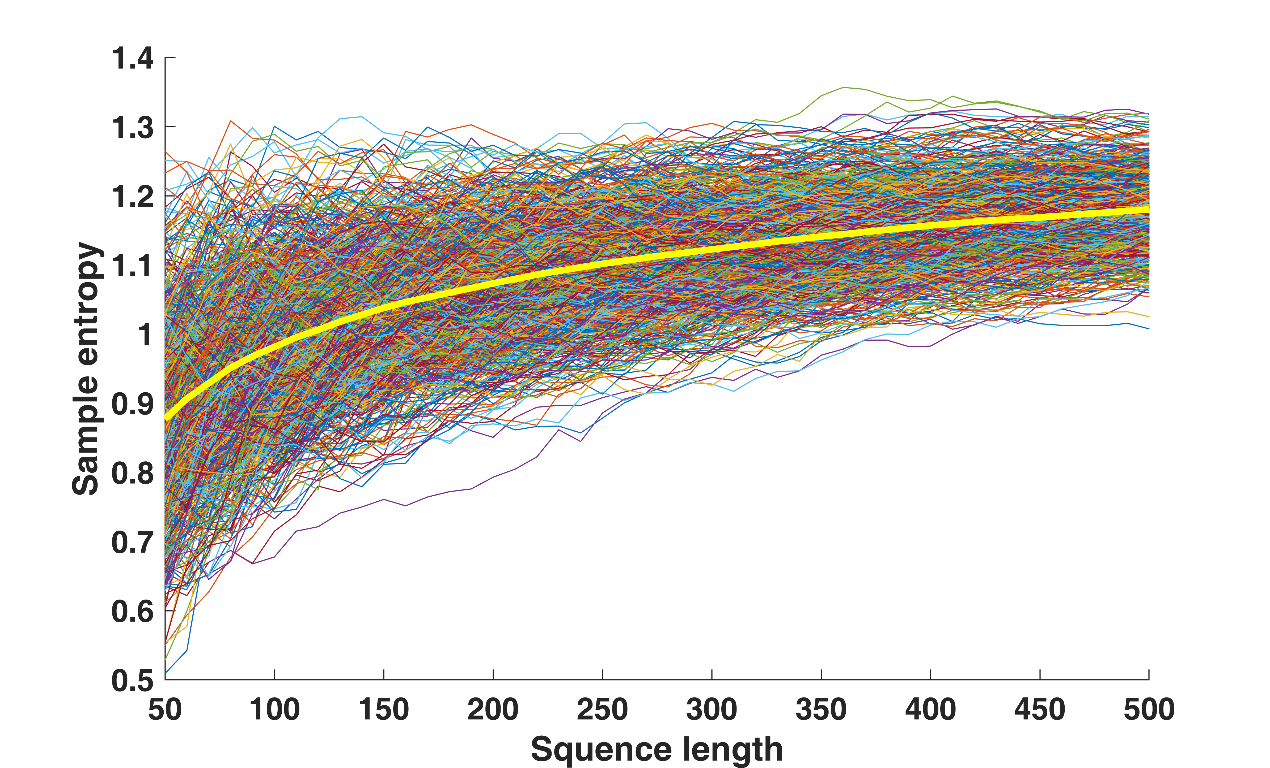
SFigure.1 Effects of sequence length on the sample entropy.**
